# Evaluating the Vitality of Introduced Woody Plant Species in the Donetsk–Makeyevka Urban Agglomeration

**DOI:** 10.3390/plants14203160

**Published:** 2025-10-14

**Authors:** Vladimir Kornienko, Inna Pirko, Besarion Meskhi, Anastasiya Olshevskaya, Victoriya Shevchenko, Mary Odabashyan, Svetlana Teplyakova, Anna Vershinina, Arina Eroshenko

**Affiliations:** 1 Scientific Research Laboratory for Monitoring and Forecasting of Donbass Ecosystems, Donetsk State University, 24 Universitetskaya St., 83001 Donetsk, Russia; pirko.i.f@mail.ru; 2 Agribusiness Faculty, Don State Technical University, 344000 Rostov-on-Don, Russia; spu-02@donstu.ru (B.M.); olshevskaya.av@gs.donstu.ru (A.O.); vshevchenko@donstu.ru (V.S.); modabashyan@donstu.ru (M.O.); steplyakova@donstu.ru (S.T.); avershinina@donstu.ru (A.V.); ppipk19@mail.ru (A.E.)

**Keywords:** urban ecosystems, environmental stress, woody plant species, vitality, functional traits

## Abstract

Introduced species of trees and shrubs used in landscaping of cities in the steppe zone are exposed to the combined negative impact of the ever-increasing load of various anthropogenic factors and unfavorable zonal natural and climatic conditions. In this regard, the assessment of the degree of plant resistance to unfavorable factors in the urban ecosystems of the steppe zone is a necessary condition for rationalizing the selection of the assortment and improving the condition of green spaces. This paper presents the results of the analysis of the vital state of 5509 representatives of 78 introduced species of trees and shrubs growing along the road and transport network in the territory with increased anthropogenic pressure. The age structure of plantings, as well as a number of biological and ecological characteristics of the species composition, are analyzed. The variation in the level of vitality in groups united by individual characteristics—taxonomic affiliation, geographical origin, morphobiological characteristics (habitus), growth rate and age of plants—is shown, and groups with the highest level of vitality are identified. As a result, a number of criteria are selected that can serve as indirect markers of plant adaptability to the ecological conditions of steppe zone cities when forming an assortment for landscaping. Using the examples of the features “plant height” and “plant age”, the species-specific reaction of plants is shown, expressed in the limitation of growth and development, as well as the reduction of life expectancy under conditions of increased anthropogenic and climatic loads. The data obtained can be used to adjust the species composition of urban trees and shrubs, optimize their ratio and spatial and functional placement, and thereby optimize the operational characteristics of green spaces and increase the duration of their use.

## 1. Introduction

Automobile transport is one of the main sources of increased environmental load in industrial cities, affecting the microclimate, air and soil pollution levels, vibration and noise pollution [1,2,3,4,5], and roadside plant communities [6,7,8,9,10,11,12,13,14,15,16]. The Donetsk–Makeevka industrial agglomeration, within which the research was conducted, is one of the largest in the Donbas region, a resource base for the coal industry. Consequently, the negative impact on the urban environment’s ecology is exacerbated by regional anthropogenic pollution characteristic of industrial regions [17,18].

In an urban context, the value of green spaces, beyond aesthetics, is determined by numerous functions, including O_2_ synthesis, phytoncide production, air ionization, adsorption of pollutants, dust retention, atmospheric CO_2_ sequestration in woody biomass, ozone layer restoration, phytoremediation, wind protection, snow retention, erosion control, precipitation regulation, noise abatement, thermoregulation, etc. [19,20,21,22,23]. However, the purification processes in the lower layers of the atmosphere lead to negative consequences for the plants themselves: dust and harmful substances accumulating on the surface of leaves disrupt photosynthesis and metabolism; dissolved heavy metals entering the internal tissues of the plant through the stomata cause chemical burns and contribute to overheating of the leaf blade in summer [12,24,25,26]. In addition, various mechanical effects (destruction of roots during construction work, vibration processes, disruption of the development of the root system, etc.) also inhibit the health of plants depending on the degree of their resistance. All these factors taken together reduce the viability of green spaces [13,14,27,28,29,30,31,32].

One of the problems of phytooptimization of urban ecosystems with increased technogenic load is the discrepancy between the species composition of green spaces and modern ecological and economic requirements. In most cases, the balance between native and introduced species is disturbed in favor of the latter in landscaping of industrial cities. Specifically, in the roadside plantings of the study area, native species account for only 30% of the floristic composition. A broader introduction of representatives of the local dendroflora is undoubtedly advisable and promising. However, given the natural limitation (paucity) of the woody plant species composition in the steppe zone where the study area is located, the use of introduced species in urban landscaping is inevitable.

Introduced species in the green infrastructure of the urban environment present a range of advantages and disadvantages. Many of them have undeniably high aesthetic qualities and functional characteristics. However, some of them, including species that are ecologically flexible and easily adaptable to new conditions, pose a threat of uncontrolled spread and invasion into anthropogenically transformed and even natural phytocenoses. Other introduced species, unlike native ones, are insufficiently adapted to regional edaphic and gradually changing meteorological and climatic conditions. As a result, their viability and, consequently, their functional longevity are reduced [7,16,33], which increases the maintenance costs for green spaces. Furthermore, operational safety is diminished, as severely compromised plants, particularly older ones, are prone to windthrow, which can damage roadside utilities and vehicles, and in some cases, lead to loss of life.

In this regard, this paper analyzes the state of introduced species of trees and shrubs in urban ecosystems under the conditions of complex impact of climatic and anthropogenic factors in order to develop rational approaches to the selection of assortment and its more efficient use for phytooptimization of habitats.

## 2. Results and Discussions

According to the floristic composition, urban woody plant species are represented by two fractions—species of native flora and introduced species. In roadside plant communities, in addition to decorative and protective artificial plantings, spontaneous vegetation is observed in certain areas, which includes such species as *Acer negundo* L., *Ailanthus altissima* (Mill.) Swingle, *Robinia pseudoacacia* L., *Prunus mahaleb* L., *P. tomentosa* Thunb., *Parthenocissus quinquefolia* (L.) Planch, and others. The total number of trees and shrubs in the 13.3 km long area is 7580 specimens, of which 5509 (73%) are introduced plants, covering 78% of the total species diversity.

**Figure 1:**
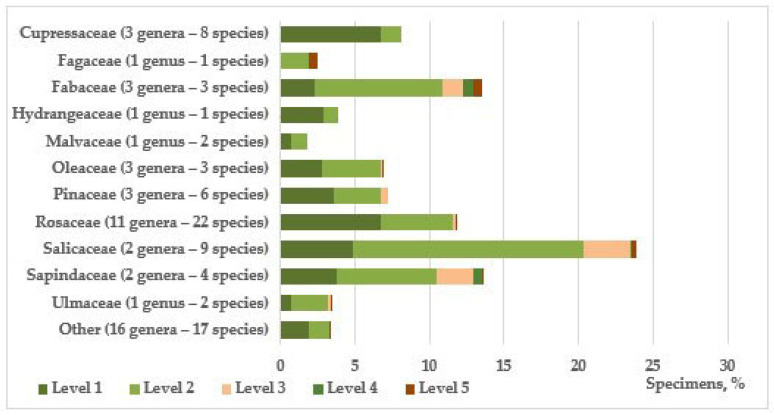
Representation and condition of trees and shrubs in roadside plantings by taxonomic groups.

The composition of introduced species is quite extensive and includes 78 species from 48 genera and 25 families, 16 of which are represented by cultivars. The families that predominate in terms of the number of species are Rosaceae (22 species), Salicaceae (9), and Cupressaceae (8), and by quantity, Salicaceae (1326 individuals), Sapindaceae (748), Fabaceae (740), Rosaceae (512) and Cupressaceae (445) (Figure 1); 43 species are represented by individual individuals or small groups (1–30). Among the genera, the dominant ones are *Populus* L. (6 species and one form—1300 individuals), *Juniperus* L. (6–353), *Prunus* L. (5–66), *Malus* P. Mill. (4–96); 31 genera are represented by single species, the rest by two or three.

In comparison, the native flora is dominated by representatives of the tree-shrub species of the family Rosaceae and, to a lesser extent, Fabaceae. It also features the families Sapindaceae, Fagaceae, Pinaceae, Malvaceae, Oleaceae, Cornaceae, Berberidaceae, Betulaceae, and Viburnaceae. Other families (Salicaceae, Cupressaceae, Hydrangeaceae, Juglandaceae, Simaroubaceae, Scrophulariaceae, Paeoniaceae, Vitaceae, Platanaceae, Rutaceae, Taxaceae, Ulmaceae) for the Eastern European province, within which the study area is located, are representatives of phylogenetically close regions (North American, Mediterranean, East Asian), which determines the relative success of the introduction of species of these taxonomic groups (Figure 1). Additional confirmation of this is the analysis of the geographic distribution of the studied species of introduced trees and shrubs (Table 1).

It was revealed that the most represented among the introduced species, both in number of species and individuals, are Eurasian and North American species, introduced on the principle of phylogenetic kinship and phytoclimatic analogues. However, based on the results of assessing the level of vitality of plants representing different geoelements, i.e., groups of species with the same general distribution, confined to the zonal type of vegetation, it was shown that the highest percentage of healthy individuals, in all age categories, was noted in *Aesculus hippocastanum* L., *A. altissima*, *Malus floribunda* Siebold ex Van Houtte, *Morus alba* L., *Populus nigra* f. *italica* (Münchh.) A. Andersen, *P. simonii* Carrière, and *Ulmus parvifolia* Jacq., i.e., East Asian and Mediterranean species, the bioecological characteristics of which best correspond to regional natural and climatic conditions, characterized by gradual aridization (Table 1).

**Figure 2:**
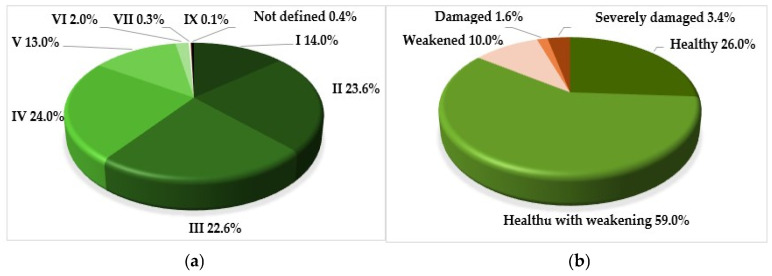
Percentage of introduced trees: (**a**) by age classes (age range, years): I—up to 10; II—11–20; III—21–30; IV—31–40; V—41–50; VI—51–60; VII—61–70; IX—81–90; (**b**) by the vital state of stands.

The age structure of the plantations, from a functional point of view, is quite balanced (Figure 2). In general, 62.3% of the plantations consist of reproductive individuals, which most effectively perform decorative and protective functions.

Despite the fact that the overwhelming majority of trees in the plantations are no older than 40 years, the proportion of healthy individuals among them is only 26% (Figure 2).

**Figure 3:**
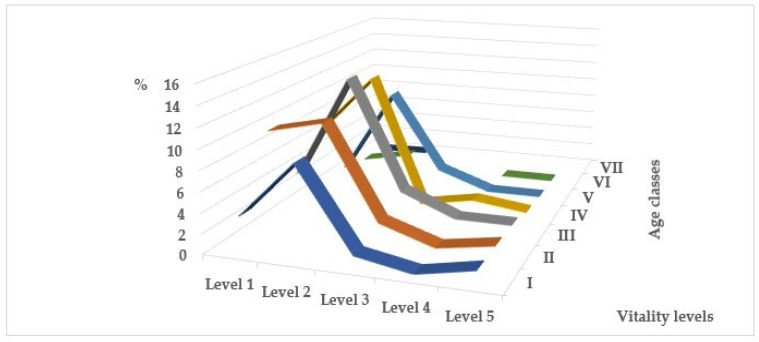
Viability level of introduced tree species in different age classes: I—up to 10; II—11–20; III–21–30; IV—31–40; V—41–50; VI—51–60; VII—61–70.

**Figure 4:**
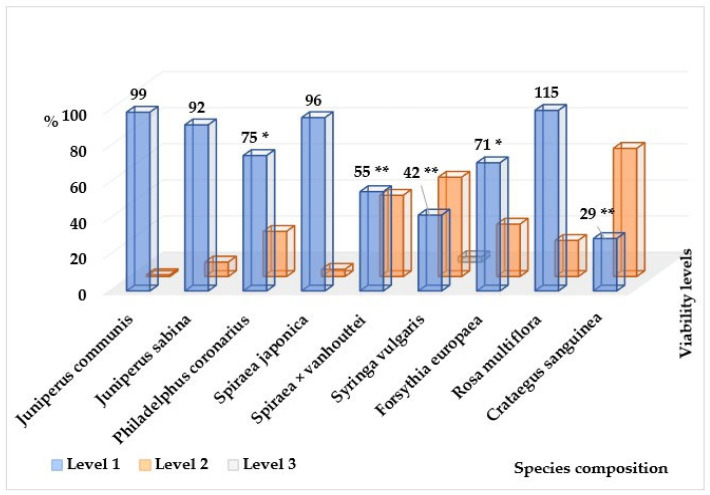
Distribution of the number of individuals of the most represented introduced shrub species by vitality levels. *—the differences are significant (*p* level ≤ 0.05); **—the differences are significant (*p* level ≤ 0.01).

In all age groups of trees, the number of plants weakened to one degree or another is greater than the number of healthy ones (Figure 3). There are violations of the crown density and its foliage, discrepancies between the size and color of leaves/needles and the species characteristics and ages of the individuals, inhibition of apical growth with a change in the typical shape of the crown, dry tops and/or drying of skeletal branches in the crown, violations of the integrity of the bark and bast, infection by pathogens, damage by pests, and changes caused by other natural and anthropogenic environmental factors. Weakening of plants and slowing down of growth processes are observed already at an early age, at the juvenile stage, and initial stages of generative development, when biological prerequisites for aging and death of plants are absent. The degree of changes largely depends on the proximity of plants to the road surface, and, consequently, on the level of intensity of anthropogenic load. As a result of comparisons of biological, habitual, and ecological characteristics of individual species [9,10,21,23,24,25,26,34,35,36,37,38,39,40,41,42,43,44,45,46] and the obtained experimental data on the mechanical resistance and maximum height of mature individuals and life expectancy in roadside conditions [6,7,16,30,31,32,33,47,48,49], it was shown that the degree of oppression is quite high and largely species-specific (Table 2). Reduction in the size of introduced species of trees and shrubs in urban conditions is a consequence of disruption of normal growth and development processes under the influence of unfavorable factors, and can be considered as one of the forms of adaptation, i.e., restructuring of homeostatic equilibrium at a new level. This is confirmed by the data we obtained, indicating a higher level of vitality of low-growing forms. The degree of height changes ranges from 0 to 75%. But on the other hand, such a biomass deficit under the influence of environmental stress leads to a significant reduction in life expectancy by 20–98.5% compared to places of natural distribution of introduced species. Trees in roadside plantings do not survive to the last ontogenetic stages (old generative (g3) and senescent (s) plants). This trend indicates high pressure created by a combination of negative environmental factors. A different picture is observed among shrubs, in plantings of which the number of healthy plants is 71% (Figure 4). Among young plants aged 5–10 years, healthy ones make up 62%, and among plantings aged 15–20 years and older, 98%.

**Figure 5:**
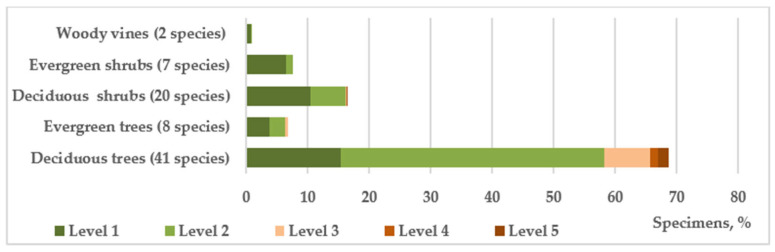
The vital state of introduced trees and shrubs of different habits in roadside plantings.

According to the complex of morphological and functional characteristics, including the features of seasonal development, the introduced species used in roadside plantings represent 5 habitual groups (Figure 5). It is shown that deciduous trees (41 species—68.7% of individuals) and shrubs (19 species—16.32% of individuals) predominate in terms of species composition and number of individuals. It should be noted that the level of viability, and therefore, adaptability to the conditions of roadside strips of all shrubs, as well as coniferous evergreen trees, is higher than that of deciduous trees dominating in the plantings. Shrubs evolutionarily have a more perfect phenotypic system of adaptation to fluctuating and/or sharply changing environmental conditions, which allows restoring the internal dynamic equilibrium to a new level at the early stages of development [39]. In general, the share of shrubs in the study area is 24%, i.e., their ratio to tree species is at the level of 1:3, whereas when landscaping roadside areas in the steppe zone, their ratio, given their higher viability and functionality, should be higher [36]. Such an imbalance increases the anthropogenic load on tree species, negatively affecting their viability and lifespan. In roadside plantings, shrubs are characterized by high functional properties and retain soil moisture, accumulate humus, and protect the fertile layer from erosion, road surfaces from snow drifts, and adjacent areas from the negative impact of motor vehicles, effectively reducing noise levels (up to 25 dB) and air velocity in the ground layer, trapping a large amount of dust [39].

**Figure 6:**
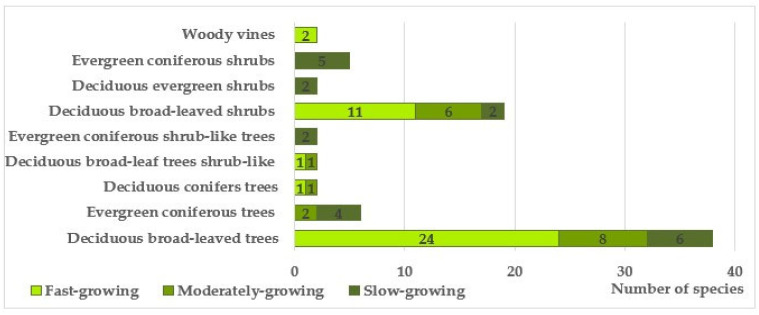
Distribution of introduced species of trees and shrubs in roadside plantings by habit and growth rates.

Based on a complex of traits, including morphological (deciduous, coniferous), habit-based (trees, shrubby trees, shrubs, lianas), and phenorhythmotypic (deciduous, evergreen), the studied species were grouped into 9 categories (Figure 6). It was shown that deciduous trees and shrubs dominate in terms of both species composition and abundance of individuals.

Among them are the species that, according to a number of authors [21,40,41,42,50,51], most intensively saturate the atmosphere with oxygen and effectively perform noise, gas, and dust protection functions: *A. negundo*, *Populus balsamifera* L., *P. nigra* f. *italica* (Münchh.) A. Andersen, *U. laevis*, *R. pseudoacacia*, *Crataegus sanguinea* Pall. and *Philadelphus coronarius* L. In roadside plantings, deciduous plants, especially species that retain dust particles well on the leaf surface and/or accumulate toxic substances absorbed from the air and soil in the leaf tissues, have a significant functional advantage compared to other species of life forms, since, provided that leaf litter is guaranteed to be removed outside the city with subsequent bioremediation, they help cleanse the road network and adjacent areas of toxicants, as well as pathogenic microorganisms and plant pests.

Coniferous trees, which are the most active producers of phytoncides that help reduce pathogens in the ground layer of air, are represented by 15 species and account for 15.9% of the total number of individuals, most of which are evergreen trees and shrubs (13 species—14.8% of individuals). Coniferous evergreen trees and shrubs growing in plantations (*Picea pungens* Engelm, *Thuja occidentalis* L., *Juniperus sabina* L., *Juniperus communis* L.) also perform a good dust-retaining function, including in winter, generally retaining more dust than broad-leaved species during the growing season [43]. Thus, these groups, characterized by high functional potential, have specific features that, for their more effective use, must be taken into account at the stage of spatial and architectural planning for roadside green zones.

When studying the distribution of tree and shrub species by growth rate, it was revealed that fast-growing species predominate in the plantations (64.9%) (Figure 6).

**Figure 7:**
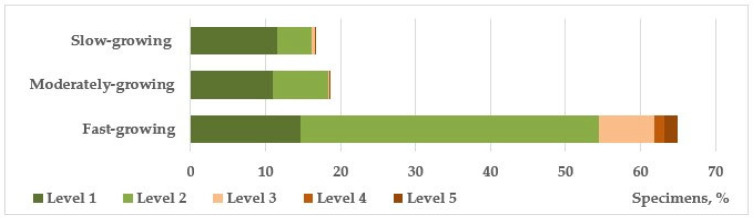
The vital state of introduced tree and shrub species of different growth rates.

Among the trees that form the main framework of linear plantings, fast-growing species also quantitatively dominate, making up 78%. The ratio of healthy and weakened plants is 18% to 82%, respectively. In the previous series of studies on the mechanical stability of introduced trees in urban landscaping of the Donetsk–Makeyevka agglomeration, it was revealed that fast-growing species are less resistant to abiogenic mechanical effects characteristic of the natural and climatic conditions of the steppe zone (frequent strong gusts of wind, snow accumulation, icing) [30,32,33,47], which is confirmed by the data we obtained (Figure 7). Medium- and slow-growing groups are represented mainly by conifers and flowering plants, which perform a predominantly decorative function and, with rare exceptions, are not planted in close proximity to the road surface, unlike fast-growing species.

**Figure 8:**
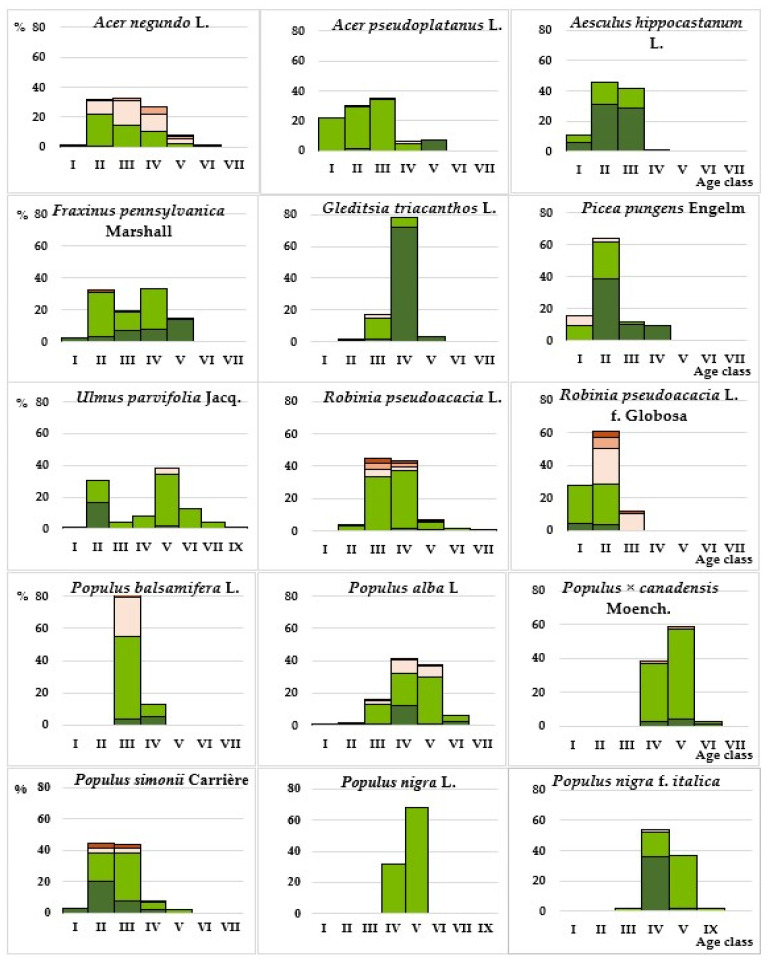
Distribution of the most represented woody introduced species by age classes and vitality levels Legend: vitality levels: 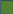
—Level 1, 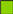
—Level 2, 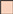
—Level 3, 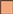
—Level 4, 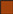
—Level 5.

In terms of taxonomic composition, 45% of introduced species growing in the study area are widely represented in roadside landscaping of cities and populated areas in the ravine-dry steppe zone, as they have been included in the recommended nomenclature lists of plants for landscaping this zone for several decades [36,42,46]. Among fast-growing species, this correspondence is 72%. In terms of quantitative representation, the basis of the studied roadside plantings is made up of 18 species, 11 of which have been growing in the study area for ≥50 years and are relatively regularly renewed in plantings (Figure 8).

A third of this assortment consists of representatives of the genus *Populus* L., which are characterized by high functional characteristics in protective plantings [44,45].

The most numerous in plantations is *P. alba* L. When studying the age structure of species of this genus, it was found that most poplars (69%) have reached the aging stage. Their average lifespan is 100–200 years; however, as practice shows (Table 2), within the city limits, the maximum safe lifespan of most species does not exceed 45–50 years, which is confirmed by more detailed studies of the condition of representatives of *P. bolleana* Lauche in urban conditions [48]. Having soft wood and sap rich in sugars, they are highly susceptible to bacterial infections that destroy the conductive and root system from the inside, which, under the influence of external factors (wind, strong vibrations), leads to the fall of large branches and the trees themselves, often without visible damage. The situation is aggravated by pruning, since the cut/sawed areas are the zone of penetration of pathogens and subsequent infection of the vascular system. Despite the fact that 14% of adult trees of the Poplar genus in the study area were found to be healthy based on visual characteristics, and 44% were found to be healthy with minor weakening, their further use carries certain risks.

The next most numerous species is *R. pseudoacacia*, plantations of which in the last 30 years have been replenished mainly with its more compact cultivar form *Globosa*. Both forms (natural and cultivated) effectively perform a protective function, but are not sufficiently resistant to mechanical impacts and are often damaged by strong gusts of wind, which further worsens their condition, so it is advisable to use them in areas most protected from strong winds.

The most numerous representatives of the *Acer* L. genus are *A. negundo* and *A. pseudoplatanus* L., the plantings of which are regularly renewed (Figure 8). One of these species, *A. negundo*, is not desirable in roadside plantings, as it is invasive [44,52,53], despite a number of bioecological features that allow it to perform a protective function quite effectively. *A. negundo* forms abundant self-seeding and numerous shoots that clog lawns and destroy asphalt, complicating or completely limiting the possibility of servicing various utility lines and collectors; its branches quickly reach overhead electrical wires [46]. It produces a large amount of allergenic pollen, is allelopathically aggressive, suppresses the growth and development of all vegetation in the lower tiers, and accelerates the process of litter mineralization [54]. Its wood is loose and fragile, so trees of this species often become hazardous. Due to the high level of invasion and other above-mentioned disadvantages, the use of this species in linear plantings along the road network is unacceptable, since the road transport network, due to its functional features, is a corridor for the active transfer of plant reproductive material. Two more introduced species used, characterized by varying degrees of invasive activity, are included in the list of invasive species [53]. One of them, which is quite well represented and constantly renewed in roadside landscaping, is *Fraxinus pennsylvanica* Marshall. It is less aggressive than *A. negundo* and most active in suburban areas, but also produces large amounts of allergenic pollen. Despite its high resistance to dust, gas, and salt, it is not wind-resistant enough and is not very decorative. *A. altissima*, represented in the study area, is currently the only plant species included in the list of “Regulated non-quarantine pests in the territory of the Russian Federation”, and is characterized by all the signs of invasiveness. In Crimea, it is a threat to natural vegetation. In the territory of the Donetsk–Makeyevka agglomeration, it is found as a weed on roadsides, spontaneous dumps, and waste heaps, i.e., it is highly resistant to man-made pressure. It is decorative during flowering, especially male individuals, which, however, have an unpleasant aroma.

Species such as *P. pungens*, *U. parvifolia*, and especially *Gleditsia triacanthos* L., in 40-year-old stands in which healthy individuals dominate, are quite stable (vitality level 1–3) (Figure 8). Due to its bioecological characteristics—drought-resistance, tolerance to environmental pollution, and salt-tolerance—*G. triacanthos* is quite promising for more widespread use, especially its thornless forms. *P. pungens* and *U. parvifolia*, which are unstable to soil salinity but are well adapted to other anthropogenic factors, can be successfully used in roadside landscaping outside of linear plantings.

Among the breeds characterized by the dominance of healthy individuals over weakened ones, *A. hippocastanum* is marked, annually attacked by the chestnut leaf miner (*Cameraria ohridella* Deschka & Dimic). Moreover, this is one of the few species whose plantings in the study area have high vitality (within 90–100% of the sample) and are classified as healthy (1–2 points). Among them, in addition to *A. hippocastanum*, there are four species of trees (*P. nigra* f. *italica*, *Malus niedzwetzkyana* Dieck., *Malus purpurea* (*A. barbier*) Rehder, *G. triacanthos*) and four species of shrubs (*Spiraea japonica* L., *J. communis*, *J. sabina*, *Ph. coronarius*). This paradoxical situation is explained by the fact that damage to leaves by the leaf miner and reduction of photosynthetic activity certainly affects the formation of fruits and annual growth, somewhat reducing the level of vitality (34% of trees in plantations are healthy with weakening) and reducing the decorative effect of plantations by mid-summer; however, damage by the chestnut moth, as a rule, does not lead to significant damage and death of plants. Every year at the beginning of the growing season, visual assessment shows that the plants have a normal passage of all phenological phases of development and a fairly high level of vitality. In the landscaping of the cities of the Donetsk–Makeyevka agglomeration, among the beautifully flowering tree species, the horse chestnut is the most spectacular, but if effective methods of pest control are not found in the near future, it is necessary to take measures to replace it with more pest-resistant species of the same genus, or with alternative species.

Despite the widespread trend in landscaping aimed at updating regional assortments at the expense of native species, the need to use introduced tree and shrub species remains the same, which is especially important for the steppe zone, where the representation of native woody plant species is insignificant. The ratio between introduced and native species should undoubtedly be shifted in favor of the latter; however, drastically minimizing their use or abandoning them entirely is impractical. Firstly, not all species of the steppe flora are sufficiently decorative, which further narrows the already limited biodiversity. Secondly, with the exception of one species—*Pinus sylvestris* L.—the native steppe flora lacks coniferous plants, which possess higher phytoncidity. Thirdly, species of the steppe dendroflora are typically low-growing trees and shrubs. Such forms, alongside low-growing introduced conifers, ornamental flowering, and decorative-leaf species, are indispensable for filling the middle and lower canopy layers [39,49]. They perform gas and dust absorption functions more effectively but cannot compete with tall introduced species in terms of the large-scale absorption of carbon dioxide and release of oxygen.

Characteristics associated with a high level of vitality can serve as criteria for the formation of an optimal range of trees and shrubs for landscaping areas with high anthropogenic load in the steppe zone.

Based on the results of the analysis of the taxonomic composition; biomorphological, phenorhythmotypical, morphofunctional, ontogenetic and botanical-geographical features; and assessment of the age structure and vital status of individuals and plantings as a whole, a number of measures are proposed to optimize the ecological state, increase the functionality, and extend the service life of roadside plantings:

1. From the plant communities of the roadside network, which is an ecological corridor for the active transfer of reproductive material of plants, exclude or limit and gradually replace *A. negundo*, *F. pennsylvanica*, and *A. altissima* due to the high invasiveness of these introduced species and the allergenicity of their pollen, which is formed in large quantities.
2. Due to the low decorative and functional value of *J. regia* and *S. myrsinifolia*, as well as the allelopathic aggressiveness of *J. regia*, exclude their use in roadside plantings.
3. In plantations, gradually replace all types of poplars with male individuals, preferably pyramidal or other compact forms. Reduce their service life and, in order to avoid accidents with large individuals, regularly monitor their condition using instrumental methods.
4. In order to increase efficiency and extend the service life of plantations, review the functional and spatial distribution of species in accordance with their bioecological characteristics, as well as their ratio in plantations, since failure to maintain the recommended balance between different life forms characterized by different functional characteristics increases the load on the main mass of trees that maximally absorb dust, anti-icing reagents, and other pollutants from the soil and air. In areas directly adjacent to the roadway, it is necessary to increase the proportion of shrubs and shrubby trees, primarily slow-growing species that are more resistant to mechanical stress, giving preference to representatives of the Rosaceae and Cupressus families, which have shown a higher degree of adaptation to anthropogenic loads in the arid conditions of the steppe zone.
5. Reproduction of plants for landscaping roadside areas should be carried out against a provocative background, i.e., in regional nurseries, in areas with a sufficiently high anthropogenic load, using material from the most viable individuals selected directly from linear plantings. From the plant communities of the roadside network, which is an ecological corridor for the active transfer of reproductive material of plants, exclude or limit and gradually replace *A. negundo*, *F. pennsylvanica*, and *A. altissima* due to the high invasiveness of these introduced species and the allergenicity of their pollen, which is produced in large quantities.

Given the state of tree and shrub plantings in roadside landscaping and in general on the territory of the Donetsk–Makeyevka agglomeration and the need to optimize environmental conditions by means of landscaping, it should be noted that the need for mass production of planting material will increase sharply. In our opinion, it is more expedient to prepare planting material in regional scientific introduction and selection centers designed to increase the adaptability of introduced species to local conditions and improve their functional traits using various selection methods and biotechnology and subsequent preparation of seed/planting material for reproduction nurseries.

## 3. Materials and Methods

The climate of the southern steppe subzone of the East European Plain, within which the study area is located, is moderately continental with arid features. The average annual precipitation is 450–550 mm. The average temperature of the coldest month of the year (January) is −6.0–(−7.8) °C; that of the warmest month (July) is 20.9–24 °C. The absolute minimum is –35 °C, the absolute maximum is 40 °C. Humidity deficit prevails, especially in July–August; due to frequent dry winds, evaporation exceeds precipitation by 200–300 mm. Snow cover is thin and unstable; its average height is 3–9 cm. Eastern and southeastern winds are characteristic, the speed of which can reach 15–20 m/s, which leads to dust storms. The period with an average daily temperature above 0 °C lasts 170–210 days [37,55].

**Figure 9:**
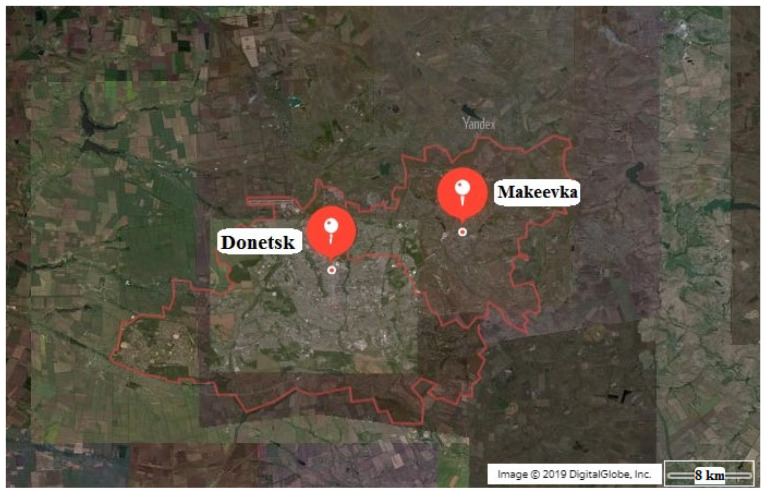
Territory of the research of the state of trees and shrubs in roadside landscaping of the Donetsk–Makeyevka agglomeration.

Studies of the condition of roadside plantings and the response of trees and shrubs to adverse environmental factors have been conducted since 2014. The survey covers a section of the road transport network in the territory of the Donetsk–Makeyevka agglomeration, 13.3 km long (Figure 9). The section is part of a regional public road (Donetsk–Makeyevka–Torez) of category II with a calculated traffic intensity of 3000 to 14,000 traffic units (TU) (with a passenger transport share of ≥30%). Geographically, the study area is located in the south of the East European Plain.

The study was carried out using the inventory method of green spaces with clarification of the taxonomic affiliation and some parameters of trees. To estimate the height of the shrubs, we used a measuring tape (at least 5 measurements from the array), and to estimate the diameter of the branches, we used a caliper (we measured the diameter of the base of at least 5 branches). To estimate the height of the trees, we used an electronic altimeter—HEC Haglof (Långsele, Sweden, 2017), and to estimate the trunk diameter at breast height (1.3 m) and the base of the skeletal branches, we used a measuring fork—Haglof Mantax Black (Långsele, Sweden, 2024). The data of the visual inspection of the studied trees were documented using a Nikon Coolpix S2600 camera (Sendai, Japan, 2009). Subsequent office processing and image analysis were performed in the AxioVision Rel. 4.8 program (Jena, Germany, 2013) using reference scaling. An additional inspection of the crown (damaged area, density, light windows) was carried out for all trees using photo fixation and further digital processing in the Axio Vision Rel. 4.8 program. The area of crown damage was determined using the software package functions based on the ratio of the total crown area to the damaged area (e.g., areas with chlorotic leaves, or leaves damaged by pests, etc.). The age of plants was determined based on archival documents from municipal services and the Donetsk Botanical Garden, which reflect the planting date and origin of the planting material, and archival photographs taking into account the growing conditions of specific individuals, as well as by using a number of direct and indirect methods, e.g., from extracted cores (using a Pressler borer), counting annual rings on cross-sections of thick (skeletal) branches. The age of shrubs was determined from cut branches as model samples (for all shrubs, at least 3 samples were selected from the array). Since the age structure of plants in the sample has a wide range (trees are represented by individuals aged 8–89 years, shrubs 4–20 years), for ease of analysis, they were grouped into age classes with an interval of 10 years for trees and 5 years for shrubs. Trees of class VIII (71–80 years) are absent from the sample.

The assessment of the level of viability of trees and shrubs was carried out on the basis of data obtained in 2023–2024. Tree vitality was assessed using V.A. Alekseev’s scale [56]:

- Level 1 (healthy tree)—there is no external damage to the crown and trunk; crown density is typical for dominant trees; dead and dying branches are concentrated in the lower part of the crown and are absent in its upper half; leaves that have finished growing are green or dark green; lifespan is typical for the region; leaf damage is insignificant (<10%) and does not affect the condition of the tree.
- Level 2 (damaged (weakened) tree)—at least one of the following signs is required: a decrease in crown density by 30%; the presence of 30% dead and (or) drying branches in the upper half of the crown; damage (nutrition, burn, chlorosis, necrosis, etc.); and exclusion of 30% of the leaf surface from assimilation activity.
- Level 3 (severely damaged (severely weakened) tree)—at least one of the following signs is required: a decrease in crown density by 60% due to premature leaf fall or thinning of the skeletal part of the crown; the presence of 60% dead and (or) drying branches in the upper half of the crown; damage by various factors and the exclusion of 60% of the leaf area from assimilating activity; the presence of the death of the top of the crown.
- Level 4 (dying tree)—the crown is destroyed; its density is at least 15–20% compared to a healthy one; >70% of branches, including in the upper half, are dry or pale green, yellowish, orange-red in color; necrosis is whitish, brown or black; signs of pest infestation are possible in the butt and middle part of the trunk.
- Level 5 (fresh and old dead wood)—dead trees. They may have remains of dry needles or leaves; the bark and small branches are often intact. As a rule, they are inhabited by xylophagous insects.

The morphobiological and bioecological traits determining the functional traits of introduced species are given according to published data [21,34,35,36].

The integral level of anthropogenic pollution of the study area comprises two components: regional, including anthropogenic pollution of the area within the Donetsk coal basin; and local, determined by the negative impact of road network management. The Donetsk coal basin has been exploited for over two hundred years, which has led to the surface extraction of large volumes of overburden and host rocks. More than 1500 dumps have formed on its territory, containing about 1400 million m^3^ of rock material, occupying more than 12 thousand hectares of the most fertile soils in the world—chernozems—and representing primary anthropogenic pollution zones. Fragments of dump rocks, from blocks to clay particles, are stored mainly in the form of waste heaps up to 80 m high [17,18]. Combustion of waste heaps emits carbon monoxide (CO), nitrogen oxides (NO_x_), sulfur dioxide (SO_2_), coal dust particulates, and heavy metals. Annual emissions from waste heaps total ~70,000 t, including CO (38,000 t), particulates (>14,000 t), and NO_x_ (>5000 t) [33,47,57]. The total level of airborne industrial pollution in the territory of the Donetsk–Makeyevka agglomeration is quite high, despite the socio-economic decline over the past 10 years, which led to a decrease in harmful emissions from industrial and transport facilities. Exceedances of Maximum Allowable Concentrations (MACs) include dust (1.4×), SO_2_ (2×), CO (3×), NO_2_ (2.5×), NH_3_ (5.5×), phenol (10×), and formaldehyde (6.6×) [22]. For soils in the zone of influence of large highways, the heavy metal concentrations exceed the MAC by 40% on average. Sound pressure levels on Ilyicha Avenue exceed permissible limits by 45–51%, with a frequency spectrum at the maximum energy of 400–800 Hz [6]. A high level of pollution was also present during the period of planting plants (1970–1990), which reached their maximum longevity in decorative and protective plantings of the study area.

The names of taxa are given in accordance with World Flora Online [58].

The Microsoft^®^ Excel^®^ LTSC MSO (version 2505, build 16.0.18827.20102) (Microsoft Corporation, Redmond, WA, USA) was used for statistical data processing.

## 4. Conclusions

The roadside plantings in the study area include 78 species of introduced trees and shrubs from 48 genera and 25 families. The presented species have varying degrees of adaptation to growing conditions—from weakly resistant to fully adapted, including invasively dangerous. The survey revealed that only 26% of introduced species are healthy, which is the result of a combination of local negative impacts of transport infrastructure, regional anthropogenic pollution of the Donetsk coal basin, and unfavorable natural and climatic conditions of the steppe zone for tree and shrub vegetation. Plants oppressed to varying degrees were found in all age groups. When assessing the level of plant viability within different families, it was found that the Cupressaceae (82% of healthy individuals) and Rosaceae (56%) are the most adapted to the natural, climatic, and anthropogenic loads in the study area.

In terms of origin, the plantations include European–Caucasian, Eurasian, Central Asian, East Asian, Mediterranean, and North American species. In terms of species composition and numbers, Eurasian and North American species dominate, but a higher level of adaptation is noted in East Asian and Mediterranean species, the proportions of healthy individuals in which are 48% and 50%, respectively.

The conducted studies have shown a certain connection between the level of plant adaptation and some biological characteristics. For example, in terms of habitual characteristics, shrubs, both evergreen (57% of healthy individuals) and deciduous (64%), as well as low-growing trees (70%), showed greater viability (*p* ≤ 0.01). When distributing species into groups by growth rate, it was shown that the most vulnerable, especially to mechanical impacts (strong gusts of wind, snow accumulation, icing, etc.), is the group of fast-growing species (23% of healthy individuals), while for moderately growing and slow-growing species, these figures are 59% and 69%, respectively (*p* ≤ 0.05).

## Figures and Tables

**Table 1:** Ratio of introduced species by geographical element of flora and levels of vitality.

Geoelement	Species		Individuals		Ratio of Individuals by Vitality Levels, %				
	N	%	N	%	1	2	3	4	5
European–Caucasian	14	18	585	11	44	54	1	-	1
Eurasian	20	25	1544	28	36	53	10	0.4	0.6
Central Asian	4	5	121	2	41	54	5	-	-
East Asian	16	21	757	14	48	47	3	-	2
Mediterranean	4	5	567	10	55	44	1	-	-
North American	20	26	1935	35	26	53	12	3	6
Total:	78	100	5509	100	-	-	-	-	-

**Table 2:** Bioecological characteristics and response of introduced species to integrated climatic and anthropogenic pressure.

Species	Resistance to Mechanical Loads [32,47]		Natural Environment		Urbanized Environment		Degree of Change, %	
	Static	Dynamic	Height, m	Longevity, Years	Height, m	MSL-UC, Years	Height, m	MSL-UC, Years
*Acer negundo*	-	-	20	90	12.1	50	−39.5	−44.4
*A. pseudoplatanus*	±	±	30	200	9.7	50	−67.7	−75
*A. saccarinum*	±	±	25	100	11.8	50	−52.8	−50
*Aesculus hippocastanum*	+	±	35	200	10.1	50	−71.1	−75
*Ailanthus altissima*	±	±	30	100	18	45+	−40	−55
*Berberis aquifolium*	+	+	1	50	ned	ned	ned	ned
*Buddleia japonica*	+	+	1.5	25	ned	ned	ned	ned
*Buxus sempervirens*	+	+	15	500	10	20+	−33.3	−96
*Caragana arborescens*	+	+	5	40	2	20+	−60	−50
*Chaenomeles japonica*	+	+	1.5	80	1	10+	−33.3	−87.5
*Cornus alba*	+	+	3	25	2	20	−33.3	−20
*Corylus colurna*	+	+	30	200	12	50+	−60	−75
*Crataegus crus-gally*	+	+	12	ned	5	ned	−58.3	ned
*Crataegus sanguinea*	-	+	8	200	6	20	−25	−90
*Crataegus. submollis*	+	+	8	ned	3	15	−62.5	ned
*Forsythia europaea*	+	+	3	70	1.5	10+	−50	−85.7
*Fraxinus pennsylvanica*	±	±	20	200	17	50	−15	−75
*Gleditsia triacanthos*	+	±	40	300	18	60	−55	−80
*Juglans nigra*	±	±	40	130	11	40	−72.5	−69.2
*Juglans regia*	+	+	30	300	10	20+	−66.7	−93.3
*Juniperus chinensis*	+	+	5	600	2	15	−60	−97.5
*Juniperus communis*	+	+	8	600	3	10+	−62.5	−98.3
*Juniperus horizontalis*	+	+	0.4	200	0.3–0.4	10+	0	−95
*Juniperus sabina*	+	+	2	500	2	15	0	−97
*Juniperus squamata*	+	+	5	400	5	10+	0	−97.5
*Juniperus virginiana*	±	+	20	200	6	20+	−70	−90
*Larix decidua*	+	+	40	500	12	20+	−70	−96
*Larix sibirica*	±	±	30	500	15	20+	−50	−96
*Lonicera tatarica*	+	+	4	80	2	15+	−50	−81.3
*Malus domestica*	±	±	10	100	5	10+	−50	−90
*Malus floribunda*	+	+	10	100	8	25	−20	−75
*Malus niedzwetzkyana*	+	+	8	100	5	20	−37.5	−80
*Malus purpurea*	+	+	8	100	6	10+	−25	−90
*Morus alba*	+	+	20	250	10	50+	−50	−80
*Parthenocissus quinquefolia*	+	+	20	ned	20	ned	0	ned
*Philadelphus coronarius*	+	+	3	40	2.5	10	−16.7	−75
*Picea abies*	+	+	35	250	12	35+	−65.7	−86
*Picea pungens*	+	+	20	350	20	60	0	−82.9
*Pinus mugo*	+	+	10	150	5	20+	−50	−86.7
*Pinus nigra ssp. pallasiana*	+	+	25	500	10	50+	−60	−90
*Platanus acerifolia*	+	+	35	400	16	50+	−54.3	−87.5
*Platycladus orientalis*	±	±	10	1000	5	15+	−50	−98.5
*Populus alba*	+	+	30	350	16	70	−46.7	−80
*P.* × *canadensis*	+	+	45	70	18	45	−60	−35.7
*P. balsamifera*	±	±	30	120	15	45	−50	−62.5
*P. nigra*	+	+	40	200	27	100	−32.5	−50
*P. nigra* f. *Italica*	+	+	40	150	20	50	−50	−66.7
*P. simonii*	+	+	20	85	16	50	−20	−41.2
*P. tremula*	+	+	20	90	ned	ned	ned	ned
*Prunus armeníaca*	-	-	12	80	8	30+	−33.3	−62.5
*Prunus cerasifera*	+	+	10	100	5	20+	−50	−80
*Prúnus doméstica*	-	-	15	25	10	15	−33.3	−40
*Prunus serotina*	-	-	20	80	9	30+	−55	−62.5
*Prunus triloba*	+	+	9	50	ned	ned	ned	ned
*Ptelea trifoliata*	±	±	8	70	2	15	−75	−78.6
*Pyrus domestica*	±	±	30	200	12	25	−60	−87.5
*Quercus rubra*	+	+	30	350	15	50	−50	−85.7
*Robinia pseudoacacia*	+	-	25	300	17	90	−32	−70
*Rosa multiflora*	+	+	3	25	3	15+	0	−40
*Salix alba* f. *pendula*	±	±	30	100	8	40	−73.3	−60
*Salix caprea*	±	±	10	30	5	15+	−50	−50
*Salix myrsinifolia*	+	+	6	25	ned	ned	ned	ned
*Scandosorbus intermedia*	+	+	15	85	8	20	−46.7	−76.5
*Sorbaria sorbifolia*	+	+	2.5	25	2	10	−20	−60
*Sorbus aucuparia*	±	±	9	100	6	15+	−33.3	−85
*Spiraea* × *billiardii*	+	+	2.5	20	2.5	8+	0	−60
*Spiraea japonica*	+	+	1.5	40	1.5	10	0	−75
*Spiraea* × *vanhouttei*	+	+	2	30	1.5	10	−25	−66.7
*Symphoricarpos albus*	+	+	2	55	1	10+	−50	−81.8
*Syringa vulgaris*	+	+	8	100	3	20+	−62.5	−80
*Táxus baccáta*	±	+	5	1500	4	50+	−20	−96.7
*Thuja occidentalis*	±	+	15	100	12	30+	−20	−70
*Tilia europaea*	±	+	25	1100	15	20+	−40	−98.2
*Tilia platyphyllos*	+	+	35	200	19	60	−45.7	−70
*Ulmus laevis*	+	±	25	200	25	70	0	−65
*Ulmus parvifolia*	+	+	15	100	15	60	0	−40
*Vitis amurensis*	+	+	30	ned	ned	ned	ned	ned

MSL-UC—maximum longevity under urban conditions; ned—not enough data.

## Data Availability

The original contributions presented in the study are included in the article; further inquiries can be directed to the corresponding author.

## Abbreviations

MSL-UC	maximum longevity under urban conditions

## References

[1] Bao L., Ma K., Xu X., Yu X. (2019). Foliar particulate matter distribution in urban road system of Beijing, China. Chin. Geogr. Sci..

[2] Jacyna M., Wasiak M., Lewczuk K., Karoń G. (2017). Noise and environmental pollution from transport: Decisive problems in developing ecologically efficient transport systems. J. Vibroeng..

[3] Zinicovscaia I., Safonov A., Kravtsova A., Chaligava O., Germonova E. (2024). Neutron activation analysis of rare earth elements (Sc, La, Ce, Nd, Sm, Eu, Tb, Dy, Yb) in the diagnosis of ecosystems of Donbass. Phys. Part. Nucl. Lett..

[4] Kucherova A.V., Minnikova T.V., Kolesnikov S.I., Khrapai E.S., Nalivaychenko A.A., Sherstnev A.K. (2025). Assessment of the health of soils polluted by municipal solid waste landfill. J. Hazard. Mat. Adv..

[5] Tsepina N.I., Minnikova T.V., Kolesnikov S.I., Minkina T.M. (2024). Pollution of silver and silver nanoparticles in the ecosystems and their interactions with plants and soil microbiota. Emerging Contaminants: Sustainable Agriculture and the Environment.

[6] Kornienko V.O. (2024). Retrospective analysis of anthropogenic pollution of the city of Donetsk. Vibration and acoustic noise. Bull. Donetsk Natl. Univ. Ser. A Nat. Sci..

[7] Korniyenko V.O., Kalaev V.N. (2022). Impact of natural climate factors on mechanical stability and failure rate in Silver birch trees in the city of Donetsk. Contemp. Probl. Ecol..

[8] Celik A., Kartal A., Akdogan A., Kaska Y. (2005). Determining the heavy metal pollution in Denizli (Turkey) by using *Robinia pseudoacacia* L.. Environ. Int..

[9] Černiauskas V., Varnagirytė-Kabašinskienė I., Čėsnienė I., Armoška E., Araminienė V. (2025). Response of Tree Seedlings to a Combined Treatment of Particulate Matter, Ground-Level Ozone, and Carbon Dioxide: Primary Effects. Plants.

[10] Hofman J., Bartholomeus H., Calders K., Van Wittenberghe S., Wuyts K., Samson R. (2014). On the relation between tree crown morphology and particulate matter deposition on urban tree leaves: A ground-based LiDAR approach. Atmos. Environ..

[11] Honour S.L., Bell J.N.B., Ashenden T.W., Cape J.N., Power S.A. (2009). Responses of herbaceous plants to urban air pollution: Effects on growth, phenology and leaf surface characteristics. Environ. Pollut..

[12] Muthu M., Gopal J., Kim D.-H., Sivanesan I. (2021). Reviewing the Impact of Vehicular Pollution on Road-Side Plants—Future Perspectives. Sustainability.

[13] Rai P., Mishra R.M. (2013). Effect of urban air pollution on epidermal traits of road side tree species, *Pongamia pinnata* (L.). Merr. J. Environ. Sci. Toxicol. Food Technol..

[14] Swami A. (2018). Impact of Automobile Induced Air Pollution on road side vegetation: A Review. Int. J. Environ. Rehabil. Conserv..

[15] Yılmaz F., Aksoy Y. (2009). Şehir içi yol bitkilendirmelerinin İstanbul İli Beyoğlu İlçesi Cumhuriyet, Halaskargazi ve Büyükdere Caddesi örneğinde irdelenmesi. J. Yaşar Univ..

[16] Kornienko V.O., Reutskaya V.V. (2025). *Populus* L. trees in the urbanized environment of Donetsk. Probl. Ecol. Nat. Prot. Technol. Reg..

[17] Zakrutkin V.E., Gibkov E.V. (2016). Technogenic geochemical flows of coal mining areas and their impact on the environment (on the example of the Donetsk Basin). News Univ. North Cauc. Reg. Nat. Sci..

[18] Zakrutkin V.E., Zubova L.G., Gibkov E.V., Zubov A.R., Vorobiev S.G. (2017). Waste dump of the coal-mining areas of Donbass as source of impact on the environment. News Univ. North Cauc. Reg. Nat. Sci..

[19] Oksanen E., Kontunen-Soppela S. (2021). Plants have different strategies to defend against air pollutants. Curr. Opin. Environ. Sci. Health.

[20] Pataki D.E., Alberti M., Cadenasso M.L., Felson A.J., McDonnell M.J., Pincetl S., Pouyat R.V., Setälä H., Whitlow T.H. (2021). The benefits and limits of urban tree planting for environmental and human health. Front. Ecol. Evol..

[21] Roy R. (2019). Stress Physiology of Woody Plant.

[22] Turner-Skoff J.B., Cavender N. (2019). The benefits of trees for livable and sustainable communities. Plants People Planet.

[23] Yan A., Wang Y., Tan S.N., Mohd Yusof M.L., Ghosh S., Chen Z. (2020). Phytoremediation: A Promising Approach for Revegetation of Heavy Metal-Polluted Land. Front. Plant Sci..

[24] Keskin N., Ili P. (2012). Investigation of particular matters on the leaves of *Pinus nigra* Arn. subsp. *pallasiana* (Lamb.) Holmboe In Denizli (Turkey). Pak. J. Bot..

[25] Leghari S.K., Zaidi M. (2013). Effect of air pollution on the leaf morphology of common plant species of Quetta city. Pak. J. Bot..

[26] Mohajerani A., Bakaric J., Jeffrey-Bailey T. (2017). The urban heat island effect, its causes, and mitigation, with reference to the thermal properties of asphalt concrete. J. Environ. Manag..

[27] Ghani M.A., Stokes A., Fourcaud T. (2009). The effect of root architecture and root loss through trenching on the anchorage of tropical urban trees (*Eugenia grandis* Wight). Trees.

[28] He H.R., Hou T.-Z., Li Y.-F., Li B.-M. (2014). Advances in Effects of Sound Waves on Plants. J. Integr. Agric..

[29] Kosmala M., Roslon-Szerynska E., Suchocka M. (2008). Influence of mechanical damage on the condition of trees. Hortic. Landsc. Archit..

[30] Kornienko V.O., Yaitsky A.S. (2024). Mechanical stability of Fagus sylvatica L. in the conditions of the south of the East European Plain: The theory of loss of stability. Samara J. Sci..

[31] Kornienko V.O., Kalaev V.N. (2024). Viability of pedunculate oak in the conditions of the city of Donetsk. Sib. J. For. Sci..

[32] Kornienko V.O., Kalaev V.N. (2024). Mechanical stability of Virginian juniper trees in steppe zone of the eastern-european plain. Lesovedenie.

[33] Kornienko V.O., Kalaev V.N. (2021). Ecological and Biological Features and Mechanical Stability of Woody Plants Used in Landscaping of Donetsk City.

[34] Šerá B. (2017). Salt-tolerant trees usable for Central European cities—A review. Hort. Sci..

[35] Gałuszka A., Migaszewski Z.M., Podlaski R., Dołegowska S., Michalik A. (2011). The influence of chloride deicers on mineral nutrition and the health status of roadside trees in the city of Kielce, Poland. Environ. Monit. Assess..

[36] Sokolskaya O.B., Vergunova A.A., Tokareva V.M. (2020). Urban Greening in Forest Steppe and Steppe Zones of Russia: Solving the Problems. Sci. Res. Innov..

[37] Netsvetov M., Sergeyev M., Nikulina V., Korniyenko V., Prokopuk Y. (2017). The climate to growth relationships of pedunculate oak in steppe. Dendrochronologia.

[38] Bulygin N.E., Yarmishko V.T. (2000). Dendrology.

[39] Götmark F., Götmark E., Jensen A.M. (2016). Why be a shrub? A basic model and hypotheses for the adaptive values of a common growth form. Front. Plant Sci..

[40] Kentbayeva B., Baigazakova Z., Baybatshanov M., Asemkulova G., Kentbayev Y. (2022). Environmental Assessment of Dust-Holding and OxygenProducing Productivity of Hawthorns in Kazakhstan. Online J. Biol. Sci..

[41] Marosz A., Nowak J.S. (2008). Effect of salinity stress on growth and macroelements uptake of four tree species. Dendrobiology.

[42] Solomentseva A.S., Kolmukidi S.V., Lebed N.I., Lebed M.B., Mezhevova A.S., Berestneva Y.V., Bikmetova K.R., Isakov A.S. (2020). Tree-shrub species promising for protective afforestation and planting in the Volgograd region. IOP Conf. Ser. Earth Environ. Sci..

[43] Zeybert E.A., Akinshina N.G., Mitusov A.V. (2022). Dust Retaining Capacity of Deciduous and Coniferous Trees in Tashkent City, Uzbekistan. Cent. Asian J. Water Res..

[44] Giniyatullin R.K., Zaitsev G.A. (2022). Evaluation of the metal content of cleaned and dirty balsam poplar leaves in industrial pollution conditions. IOP Conf. Ser. Earth Environ. Sci..

[45] Zalesny R.S., Zhu J.Y., Headlee W.L., Gleisner R., Pilipovi’c A., Acker J.V., Bauer E.O., Birr B.A., Wiese A.H. (2020). Ecosystem Services, Physiology, and Biofuels Recalcitrance of Poplars Grown for Landfill Phytoremediation. Plants.

[46] Vinogradova Y., Pergl J., Essl F., Hejda M., Kleunen M.V., Pyšek P. (2018). Invasive alien plants of Russia: Insights from regional inventories. Biol. Invasions.

[47] Kornienko V.O., Kalaev V.N. (2018). Mechanical Stability of Tree Species and Recommendations for Preventing Their Accidents in Urban Areas.

[48] Kornienko V., Reuckaya V., Shkirenko A., Meskhi B., Olshevskaya A., Odabashyan M., Shevchenko V., Teplyakova S. (2025). Silvicultural and Ecological Characteristics of *Populus bolleana* Lauche as a Key Introduced Species in the Urban Dendroflora of Industrial Cities. Plants.

[49] Kornienko V., Shkirenko A., Reuckaya V., Meskhi B., Dzhedirov D., Olshevskaya A., Odabashyan M., Shevchenko V., Mangasarian D., Kulikova N. (2025). *Taxus baccata* L. Under Changing Climate Conditions in the Steppe Zone of the East European Plain. Plants.

[50] Bassuk N., Deanna F.C., Marranca B.Z., Barb N. (2009). Recommended Urban Trees: Site Assessment and Tree Selection for Stress Tolerance.

[51] Gennaro M., Giorcelli A. (2019). The biotic adversities of poplar in Italy: A reasoned analysis of factors determining the current state and future perspectives. Ann. Silvic. Res..

[52] Puchałka R., Paź-Dyderska S., Jagodziński A.M., Sádlo J., Vítková M., Klisz M., Koniakin S., Prokopuk Y., Netsvetov M., Nicolescu V.-N. (2023). Predicted range shifts of alien tree species in Europe. Agric. For. Meteorol..

[53] Senator S.A., Vinogradova Y.K. (2023). Invasive Plants of Russia: Results of Inventory, Peculiarities of Distribution, and Management Issues. Biol. Bull. Rev..

[54] Nikolaeva A.A., Golosova E.V., Shelepova O.V. (2021). Allelopathic activity of *Acer negundo* L. leaf litter as a vector of invasion species into plant communities. BIO Web Conf..

[55] Drozd G.Y. (2020). Response of the urban environment to climate change in Donbass. News Automob. Road Inst..

[56] Katjutin P.N., Stavrova N.I., Gorshkov V.V., Lyanguzov A.Y., Bakkal I.J., Mikhailov S.A. (2020). Radial growth of trees differing in their vitality in the middle-aged scots pine forests in the Kola Peninsula. Silva Fenn..

[57] Khomenko Y.V., Soldatova A.S. (2015). Assessment of the problems of the Donbass waste heaps. Econ. Bull. Donbass.

[58] World Flora Online (2024). World Flora Online Plant List. http://www.worldfloraonline.org.

